# Melittin induces in vitro death of *Leishmania (Leishmania) infantum* by triggering the cellular innate immune response

**DOI:** 10.1186/s40409-016-0055-x

**Published:** 2016-01-08

**Authors:** Andreia Vieira Pereira, Gustavo de Barros, Erika Gracielle Pinto, Andre Gustavo Tempone, Ricardo de Oliveira Orsi, Lucilene Delazari dos Santos, Sueli Calvi, Rui Seabra Ferreira, Daniel Carvalho Pimenta, Benedito Barraviera

**Affiliations:** Graduate Program in Tropical Diseases, Botucatu Medical School, São Paulo State University (UNESP – Univ Estadual Paulista), Botucatu, SP Brazil; Department of Parasitology, Adolfo Lutz Institute, São Paulo, SP Brazil; Laboratory of Protozoology, Institute for Tropical Medicine, University of São Paulo (USP), São Paulo, SP Brazil; Department of Animal Production, School of Veterinary Medicine and Animal Husbandry, São Paulo State University (UNESP – Univ Estadual Paulista), Botucatu, SP Brazil; Center for the Study of Venoms and Venomous Animals (CEVAP), São Paulo State University (UNESP – Univ Estadual Paulista), Rua José Barbosa de Barros, 1780, 18610-307 Botucatu, SP Brazil; Laboratory of Biochemistry and Biophysics, Butantan Institute, São Paulo, SP Brazil

**Keywords:** Melittin, *Apis mellifera*, *Leishmania*, Leishmaniasis, Peptides, Toxins, Antiparasitic, Cytokines

## Abstract

**Background:**

*Apis mellifera* venom, which has already been recommended as an alternative anti-inflammatory treatment, may be also considered an important source of candidate molecules for biotechnological and biomedical uses, such as the treatment of parasitic diseases.

**Methods:**

Africanized honeybee venom from *Apis mellifera* was fractionated by RP-C18-HPLC and the obtained melittin was incubated with promastigotes and intracellular amastigotes of *Leishmania (L.) infantum.* Cytotoxicity to mice peritoneal macrophages was evaluated through mitochondrial oxidative activity. The production of anti- and pro-inflammatory cytokines, NO and H_2_O_2_ by macrophages was determined.

**Results:**

Promastigotes and intracellular amastigotes were susceptible to melittin (IC_50_ 28.3 μg.mL^−1^ and 1.4 μg.mL^−1^, respectively), but also showed mammalian cell cytotoxicity with an IC_50_ value of 5.7 μg.mL^−1^. Uninfected macrophages treated with melittin increased the production of IL-10, TNF-α, NO and H_2_O_2_. Infected melittin-treated macrophages increased IL-12 production, but decreased the levels of IL-10, TNF-α, NO and H_2_O_2_.

**Conclusions:**

The results showed that melittin acts in vitro against promastigotes and intracellular amastigotes of *Leishmania (L.) infantum*. Furthermore, they can act indirectly on intracellular amastigotes through a macrophage immunomodulatory effect.

## Background

In developing countries, neglected parasitic diseases have resulted in high mortality and morbidity, especially in tropical regions. Leishmaniasis remains an important neglected tropical disease that affects 12 million people in 98 countries with 20,000 to 40,000 deaths due to visceral leishmaniasis (VL) annually [1, 2]. VL is a potentially fatal disease that has emerged as an important opportunistic condition in HIV-infected patients [3]. Its treatment is not only challenging and long, but also offers a limited arsenal of drugs, most of which are toxic including antimonials, amphotericin B, pentamidine and miltefosine [4]. The need for new treatments is crucial.

Discovering new in vitro hit compounds might be considered relatively easier than crossing the animal-to-human barrier, which is still a great challenge to drug discovery programs. Bridging this gap between clinical and basic research must be encouraged by health professionals in order to discover effective treatments, especially for neglected diseases [5]. The search to develop alternative compounds, either of natural or synthetic origin, effective for both medical therapy and chemoprophylaxis, should be carefully considered [6]. Likewise, *Apis mellifera* venom may provide an important source of candidate molecules for application against parasitic diseases, even though it has already been recommended as an anti-inflammatory treatment [7–9].

Melittin, the major component of *Apis mellifera* venom, is a cytolytic peptide that may adopt different aggregation states and conformations depending on its concentration. Some of its biological effects include a surfactant action and the ability to interact and enlarge leukocytes and mast cells by presenting detergent-like effects [10–13]. In addition to its antibacterial, antifungal, antiviral and anti-inflammatory properties, melittin also promotes the induction of histamine release [13]. In relation to its antiprotozoal effect, this peptide affects the viability and ultrastructure of trypanosomatids, including amastigotes, at concentrations approximately 100 times lower than that in mammalian cells [7]. Since *Leishmania* can exhibit apoptosis induced by antimicrobial peptides, melittin may follow the same path and then modulate cell death [14]. Acylated synthetic cecropin A-melittin hybrid appeared to be safe and effective for treating canine leishmaniasis [15]. In this context, the present study aimed to evaluate for the first time the effect of melittin as a leishmanicidal agent against *L. infantum* intracellular amastigotes. Therefore, we investigated the production of pro- and anti-inflammatory cytokines as well as oxygen and nitrogen metabolites in order to assess whether this peptide would directly act on parasites, or if it would modulate the immune response.

## Methods

### Venom collection

Venom from *Apis mellifera* Africanized honeybees (AHBs), aged between 30 and 40 days, was extracted by electrical stimulation, twice per week, from December 2010 to June 2011. The apiaries are located at the Nucleus of Teaching Science and Technology in Rational Apiculture (NECTAR) of the School of Veterinary Medicine and Animal Husbandry, UNESP, located on the Edgardia Experimental Farm, Botucatu, SP, Brazil, with the following geographic coordinates: 22°49′ South (latitude); 48°24′ West (longitude) and average altitude of 623 m [16, 17].

### Isolation and purification of melittin by high performance reversed-phase liquid chromatography (RP-HPLC)

The isolation and purification of melittin was conducted by RP-HPLC employing a Shimadzu 20A Proeminence binary HPLC system and C18 column. The lyophilized whole venom was solubilized in 0.1 % (v/v) trifluoroacetic acid at the concentration of 1 mg.mL^−1^. The samples were centrifuged and the supernatant was separated for chromatographic analysis. For fractioning, a two-solvent system was utilized: (A) H_2_O: Trifluoroacetic acid (TFA) (1:1000) and (B) acetonitrile (ACN):H_2_O:TFA (900:100:1). The elution was performed at a constant flow of 3.5 mL.min^−1^ under a linear gradient of B (5–60 %) for 15 min and the elution was monitored at 214 nm. The peptide elution was analyzed by mass spectrometry [16, 18].

### Mass spectrometry

LC-MS analyses were performed using an electrospray-ion trap-time of flight (ESI-IT-TOF) (Shymadzu Co., Japan) mass spectrometer equipped with a binary ultra-fast liquid chromatography system (UFLC) (20A Prominence, Shimadzu Co., Japan). Samples were dried, suspended in water/formic acid (0.99/0.01, v/v) and loaded in a C18 column (Shimadzu-pack XR-ODS, 2.2 μm; 100 × 3 mm) in a binary solvent system: (A_2_) water/formic acid (FA) (999/1, v/v) and (B_2_) ACN/water/FA (900/99/1, v/v/v). The column was eluted at a constant flow rate of 0.2 mL.min^−1^ with a 0 to 100 % gradient of solvent B_2_ for 20 min. The eluates were monitored by a Shimadzu SPD-M20A PDA detector before introduction into the mass spectrometer, in which the spray voltage was kept at 4.5 kV, the capillary voltage at 1.76 kV and the temperature at 200 °C. MS spectra were acquired under positive mode and collected in the 80 to 2000 *m*/*z* range. Instrument control, data acquisition, and data processing were performed with LabSolutions (LCMSsolution 3.60.361 version, Shimadzu). Direct mass spectrometric analyses were performed in an ESI-IT-TOF as described above. Samples were dried and suspended in 0.1 % FA by positive mode electrospray ionization (ESI+). The fractions were manually injected in a Rheodyne injector, at a flow rate 50 μL/min, in 50 % B_2_. Instrument control, data acquisition, and data processing were performed with LabSolutions (LCMSsolution 3.60.361 version, Shimadzu). Complementary mass spectrometric analyses were performed in a Shimadzu Axima MALDI-TOF/TOF instrument, under positive ionization mode and employing α-cyano as matrix.

### Experimental animals

Golden hamsters (*Mesocricetus auratus*) and BALB/c mice were obtained from the Adolfo Lutz Institute (São Paulo) and the Experimental Laboratory for Research on Tropical Diseases (LEPDT), in the Department of Tropical Diseases and Imaging Diagnosis at Botucatu Medical School (UNESP). The animals were maintained in sterile boxes lined with absorbent material, and received food and water *ad libitum.* The golden hamsters were monthly infected with spleen amastigotes to maintain the strain of *Leishmania*. The BALB/c mice were utilized to obtain the peritoneal macrophages. Animal procedures were performed in agreement with the Guide for the Care and Use of Laboratory Animals from the National Academy of Sciences. This study was approved on 07/28/2011 by the Ethics Committee on Animal Experimentation of the Botucatu Medical School, São Paulo State University (UNESP), Botucatu, SP, Brazil, under protocol number CEEA 8932011.

### Parasites and macrophages

Promastigotes of *L. infantum* (MHOM/BR/1972/LD) were maintained in M-199 medium supplemented with 10 % fetal bovine serum (FBS) and 0.25 % hemin at 24 °C. The amastigote forms were obtained by differential centrifugation of the spleen from infected hamsters. The macrophages were collected from peritoneal cavity of BALB/c mice by washing in RPMI-1640 medium supplemented with 10 % fetal bovine serum and maintained at 37 °C at a tension of 5 % CO_2_ [19].

### *Determination of* in vitro *antileishmanial activity*

To determine the 50 % inhibitory concentration (IC_50_) against the promastigote forms of *L.* (L.) *infantum*, cells were counted in a Neubauer hemocytometer and seeded at 1 × 10^6^ cells per well in 96-well microplates. The melittin and whole venom were dissolved in PBS and tested at 100 μg.mL^−1^ for melittin and 300 μg.mL^−1^ for whole venom with final respective doses of 0.04 μg.mL^−1^ and 0.14 μg.mL^−1^ [19]. Miltefosine was used as a standard drug and PBS as a negative control. The viability was determined by the colorimetric method of MTT [3- (4,5- dimetthythiazol-2-yl) – 2,5 diphenyltetiazoliumbromide] [20].

To determine the 50 % inhibitory concentration (IC_50_) against intracellular amastigotes of *L. infantum*, mice peritoneal macrophages were incubated in a 1:10 ratio of macrophages to amastigotes. Test compounds were incubated with infected macrophages for 120 h. Subsequently, the cells were observed through a light microscope. Miltefosine was used as the standard control drug. Parasite burden was determined by the number of infected macrophages out of 500 cells [21].

### Cytotoxicity against peritoneal cells

Peritoneal macrophages were incubated with whole venom and melittin, serially diluted from an initial concentration of 100 μg.mL^−1^ for melittin and of 300 μg.mL^−1^ for whole venom, for 48 h at a temperature of 37 °C in a 5 % CO_2_ incubator. Miltefosine was utilized as the standard drug. The viability of cells was determined by the MTT assay [22].

### Quantification of cytokines

Macrophages were incubated with promastigote forms of *L. infantum.* The infection process was similar to that displayed by the amastigote forms. Melittin was dissolved in PBS, being added at 0.70, 1.47 and 2.50 μg/mL. The supernatants were retrieved at 24, 48 and 72 h and maintained at −80 °C. We chose 72 h as the maximum period for evaluating levels of cytokines since their production is reduced or even lost in longer periods. Subsequently, the cytokines IL-12, IL-10, TGF-β and TNF-α were measured utilizing the Quantikine*®* HS kit (R&D Systems), according to the manufacturer’s instructions [19].

### Determination of nitric oxide production

The nitric oxide production (NO) was evaluated in supernatants that had been retrieved as previously described, by measuring nitrites (NO_2_^−^) and nitrates (NO_3_^−^). Nitrite was dosed by the Griess reaction [23]. The results were read in an ELISA reader utilizing a 540 nm filter and compared with a blank solution constituted by the Griess reaction. All measurements were performed in triplicate. The results were expressed in micromoles of NO/8 × 10^4^ cells, starting from a standard curve established in each assay, composed of known molar concentrations of NO_2_^−^, varying from 200 to 0.39 μM.

### Determination of hydrogen peroxide production

The production of hydrogen peroxide (H_2_O_2_) was determined according to the method described by Pick and Keisari [24] and adapted by Pick and Mizel [25]. In summary, the monolayers of macrophages were treated with red phenol buffer solution containing 140 mM of NaCl, 10 mM of phosphate buffer, 5.5 mM of dextrose, 0.56 mM of red phenol and 0.01 mg.mL^−1^ of strong root peroxidase type II (Sigma Chemical Co, USA) at pH 7. The plates were incubated at a temperature of 37 °C, in a 5 % CO_2_ incubator for 4 h, and the reaction was interrupted by the addition of 0.01 mL of 1 M NaOH. The absorbance was determined by an ELISA automatic reader (MD 5000, Dynatech Laboratories Inc., USA), with a 620 nm filter, using a blank solution constituted by phenol red and 1 M NaOH. All the measurements were performed in triplicate, and the results of H_2_O_2_ levels were expressed in nanomoles of H_2_O_2_/8 × 10^4^ cells, starting from a standard curve established in each assay, constituted from known molar concentrations of H_2_O_2_ in phenol buffer. Under our experimental conditions, the curve was constructed with H_2_O_2_ concentrations of 0.5, 1.0, 2.0, 4.0 and 8 nM_._

### Statistical analysis

The results were expressed as standard deviation of the mean (S.D.M.). The values obtained were submitted to analysis of variance (ANOVA), followed by the test of Tukey, when the number of groups was greater than two. Non-parametric data were expressed as median and analyzed by the test of Mann–Whitney. The entire analysis was performed by the software GraphPad Prism® Version 5.01 (GraphPad Software Inc., USA). For all the statistical tests, the significance level was considered *p* < 0.05 [26].

## Results

### Melittin purification

**Fig. 1:**
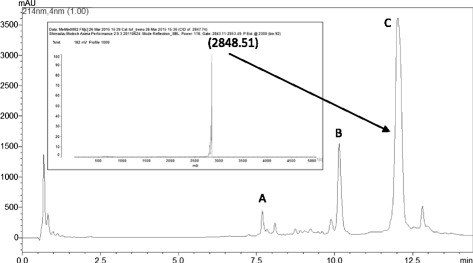
Chromatographic profile of whole venom from Africanized honeybees obtained by reversed-phase liquid chromatography indicating its major components: (**a**) apamin, (**b**) phospholipase A_2_ and (**c**) melittin. Inset: MALDI-TOF/MS profile of melittin (peak C). The m/z value is typed beside the peak for better visualization

Melittin was purified from Africanized honeybee (AHB) whole venom via reversed-phase liquid chromatography, as previously described [16–18]. Fig. 1 presents the chromatographic profile of whole venom, identifying the venom major peaks, namely: apamin (A), phospholipase A_2_ (B) and melittin (C). Peak C was analyzed by mass spectrometry (Fig. 1, inset) and demonstrated to contain pure melittin.

### Antileishmanial activity and macrophage cytotoxicity

Table 1 displays the viability of *Leishmania* promastigotes and mammalian cytotoxicity of cells challenged with whole venom, melittin and standard drugs. Melittin was active against *Leishmania* promastigotes showing an IC_50_ value of 28.29 μg.mL^−1^ (95 % confidence interval = 23.97 to 33.39 μg.mL^−1^) after 48 h. The crude venom showed an IC_50_ value of 87 μg.mL^−1^ (95 % confidence interval = 76.91 to 99.22 μg.mL^−1^); miltefosine was used as the standard drug and demonstrated an IC_50_ value of 6.69 μg.mL^−1^ (95 % confidence interval = 6.29 to 7.11 μg.mL^−1^). The statistical difference among the IC_50_ values of melittin, whole venom and miltefosine was significant (*p* < 0.05). Melittin also demonstrated cytotoxicity to mammalian cells showing an IC_50_ value of 5.73 μg.mL^−1^ (95 % confidence interval = 5.08 to 6.44 μg.mL^−1^), while the whole venom showed an IC_50_ value of 27.59 μg.mL^−1^ (95 % confidence interval = 20.63 to 36.90 μg.mL^−1^), differing significantly from melittin (*p* < 0.05). Miltefosine was also used as standard drug presenting an IC_50_ value of 49.72 μg.mL^−1^ (95 % confidence interval = 39.85 to 63.98 μg.mL^−1^) in mammalian cells.

**Table 1:** Antileishmanial and mammalian cytotoxicity of whole venom and melittin

IC_50_ (μg.mL^−1^) (95 % CI)			
	*L.* (*L.*) *infantum*		Cytotoxicity (macrophages)
	promastigotes	amastigotes	
Crude venom	87.0 (76.91 to 99.22)	n.d.	27.59 (20.63 to 36.90)
Melittin	28.29 (23.97 to 33.39)	1.40 (1.21 to 1.63)	5.73 (5.08 to 6.44)
Miltefosine	6.69 (6.29 to 7.11)	6.87 (4.71 to 10.01)	49.72 (39.85 to 63.98)

IC_50_: 50 % inhibitory concentration; 95 % CI: 95 % confidence interval; n.d.: not determined

The activity of melittin against the amastigotes was investigated using peritoneal macrophages as host cells. The results showed an IC_50_ value of 1.4 μg.mL^−1^. Although melittin was able to eliminate 100 % of intracellular amastigotes at 2.5 μg.mL^−1^, a morphological alteration of host cells could be observed when compared to control group. However, in the concentration of 0.7 μg.mL^−1^, melittin induced no toxicity to macrophages, but no considerable treatment could be detected. Miltefosine was used as the standard drug and showed an IC_50_ value of 6.87 μg.mL^−1^. Considering the selectivity index of melittin, which was determined using the mammalian cytotoxicity by the activity against intracellular amastigotes, we demonstrated a value of 4, confirming a higher toxic effect to *Leishmania* than to mammalian cells.

### Melittin effects on IL-12 and TNF-α levels

**Fig. 2:**
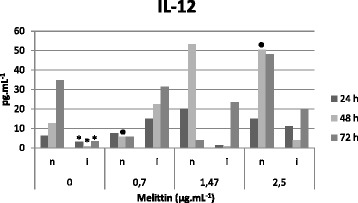
Determination of IL-12 concentration (pg.mL^−1^) in the supernatant of uninfected macrophages (**n**) and infected macrophages (**i**) after melittin treatment (0, 0.7, 1.47 and 2.5 μg.mL^−1^) at 24, 48 and 72 h. Data are presented as mean of three independent measurements. *p < 0.05 for infected cells versus non-infected cells (24, 48 and 72 h) without melittin addition. ● *p* < 0.05, for 24 h treatment with 2.5 versus 0.7 μg.mL^−1^ melittin of non-infected cells

Melittin was incubated at non-toxic concentrations with non-infected and infected macrophages while still presenting an anti-amastigote effect. This concentration was assayed for quantifying anti-inflammatory (IL-10 and TGF-β) and pro-inflammatory cytokines (IL-12 and TNF-α). In Fig. 2, we demonstrate the effect of melittin on IL-12 production. The IL-12 production levels by macrophages of the control group, which was significantly (*p* < 0.05) higher than the group of untreated infected macrophages. No significant difference was detected in IL-12 production between uninfected and melittin-treated macrophages. But it also could be noted that by decreasing melittin concentrations, the IL-12 production levels dropped. Infected macrophages treated with melittin produced increased levels of IL-12 when compared to untreated cells. A time-dependent production could be observed in longer incubation periods (72 h).

**Fig. 3:**
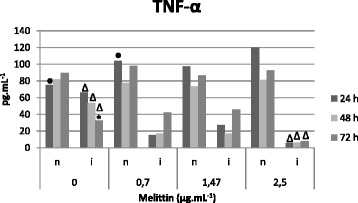
Determination of TNF-α concentration (pg.mL^−1^) in the supernatant of uninfected macrophages (**n**) and infected macrophages (**i**) after melittin treatment (0, 0.7, 1.47 and 2.5 μg.mL^−1^) at 24, 48 and 72 h. Data are presented as mean of three independent measurements. * *p* < 0.05 for 72 h infect cells versus 24, 48 and 72 h non-infected cells, without melittin addition. ● *p* < 0.05, for 24 h treatment with 0.7 μg.mL^−1^ melittin versus non-infected cells. ∆*p* < 0.05 for 24, 48 and 72 h treatment with 2.5 μg.mL^−1^ melittin versus infected non-treated cells

The TNF-α production was also determined after melittin treatment. Figure 3 shows that uninfected macrophages, independent of melittin presence, produced significant levels of TNF-α in a time-dependent manner. After 72 h of incubation, uninfected cells produced higher amounts of TNF-α (*p* < 0.05) when compared to infected cells. A significant (*p* < 0.05) reduction of TNF-α levels was observed when the infected cells were treated with 2.5 μg.mL^−1^ of melittin. Despite the observation of non-significant differences (*p* < 0.10), infected macrophages treated with melittin at 1.47 and 0.7 μg.mL^−1^ demonstrated a slight increase in TNF-α production.

### Melittin effects on IL-10 and TGF-β

**Fig. 4:**
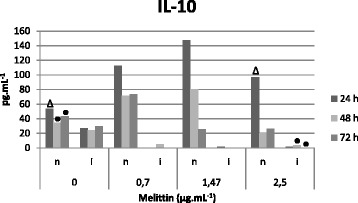
Determination of IL-10 concentration (pg.mL^−1^) in the supernatant of uninfected macrophages (**n**) and infected macrophages (**i**) after melittin treatment (0, 0.7, 1.47 and 2.5 μg.mL^−1^) at 24, 48 and 72 h. Data are presented as mean of three independent measurements. ● *p* < 0.05, for 48 and 72 h treatment with 2.5 μg.mL^−1^ melittin versus non-infected cells. ∆ *p* < 0.05 for 24 h treatment with 2.5 μg.mL^−1^ melittin versus non-infected non-treated cells. All treated infected cells (24, 48 and 72 h; 0, 0.7, 1.47 and 2.5 μg.mL^−1^) were statistically different (*p* < 0.05) of the corresponding non-treated cells

Elevated levels of IL-10 were produced by uninfected macrophages treated with melittin, particularly at 1.47 μg.mL^−1^ after the 24-h incubation (*p* < 0.05). In Fig. 4, infected melittin-treated macrophages demonstrated a significant decrease of IL-10 levels in a concentration-independent manner (*p* < 0.05). TGF-β levels showed no variation among all groups (data not shown).

### Melittin effects on NO and H_2_O_2_

**Fig. 5:**
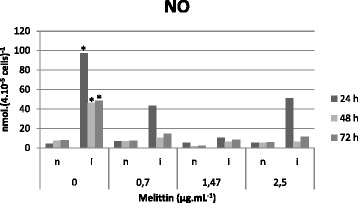
Determination of NO levels in the supernatant of uninfected macrophages (**n**) and infected macrophages (**i**) after melittin treatment (0, 0.7, 1.47 and 2.5 μg.mL^−1^) at 24, 48 and 72 h. Data are presented as mean of three independent measurements.* *p* < 0.05 for infected cells versus non-infected cells (24, 48 and 72 h) without melittin addition. All treated infected cells (24, 48 and 72 h; 0, 0.7, 1.47 and 2.5 μg.mL^−1^) are statistically different (*p* < 0.05) of the corresponding non-treated cells

**Fig. 6:**
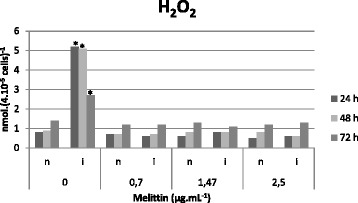
Determination of H_2_O_2_ levels in the supernatant of uninfected macrophages (**n**) and infected macrophages (**i**) after melittin treatment (0, 0.7, 1.47 and 2.5 μg.mL^−1^) at 24, 48 and 72 h. Data is presented as mean of three independent measurements.*p < 0.05 for infected cells versus non-infected cells (24, 48 and 72 h) without melittin addition. All treated infected cells (24, 48 and 72 h; 0, 0.7, 1.47 and 2.5 μg.mL^−1^) were statistically different (*p* < 0.05) of the corresponding non-treated cells

In Figs. 5 and 6, it was demonstrated that melittin did not alter levels of either NO or H_2_O_2_ in uninfected macrophages. Conversely, infected macrophages treated with melittin showed reduced NO and H_2_O_2_ levels when compared to untreated macrophages (*p* < 0.05).

## Discussion

Peptides derived from animal toxins represent an inexhaustible source of candidate compounds for drug discovery and design, resulting in a reduced probability of drug resistance through a rapid elimination of microorganisms [7]. Adade *et al*. [7] evaluated the effect of natural melittin against epimastigote, trypomastigote and amastigote forms of *T. cruzi;* the peptide was able to affect the growth, viability and ultrastructure of amastigotes at concentrations 15- to 100-fold smaller than the toxic concentrations. These effects were also observed in other microorganisms [27]. According to Klocek and Seelig [28] melittin, at 8 and 14 μg/mL, demonstrated cytotoxicity to hamster ovary cells (CHO) and also to glycosaminoglycan-deficient cells (CHO-745), respectively. Similarly, Maher and McClean [29] investigated the cytotoxicity of melittin against HT29 intestinal epithelial cells and Caco-2, with respective IC_50_ values of 3.4 μg.mL^−1^ and 5.2 μg.mL^−1^. Despite the absence in the literature of an *in vitro* toxicity of natural melittin to macrophages, our results corroborated previously published studies.

Our data demonstrated that melittin was approximately 3-fold more effective against *Leishmania infantum* promastigotes than the whole venom. Diaz-Achirica *et al.* [30] evaluated the activity of melittin against *Leishmania donovani* promastigotes and demonstrated an IC_50_ value of 0.87 μg.mL^−1^, causing damage to the plasma membrane of the parasite. In our study, *Leishmania infantum* promastigotes were about 32-fold more resistant than *L. donovani* reported above.

For decades, the effect of animal toxins on the immune system has been extensively investigated. It has been known that these toxins have the capacity to modulate the innate and adaptive immune responses [31–33]. In addition, the balance between the host and parasite factors that control the activation/deactivation of macrophages determines the outcome of the infected cells. Macrophages are the major effector cells responsible for elimination of parasites, which can be activated by distinct signals leading to their development into functionally distinct subsets with different disease outcomes. Thus, appropriate activation of macrophages is crucial for eliminating the intracellular pathogen [34]. Immune response in leishmaniasis was clearly described in the murine model as a Th2 response in active disease and a Th1 response during the elimination of infection and cure. In humans, the dichotomy is not well established when compared to the murine model, and disease progression is determined by the changing cytokine profile. Our results showed that melittin stimulated the production of interleukin IL-12 in a time- and dose-dependent manner. On the other hand, the infection diminished the levels of this cytokine, whereas melittin treatment stimulated IL-12 production at its 50 % inhibitory concentration. These results suggest that melittin may eliminate the parasite via an indirect effect, by modulating pro-inflammatory interleukin IL-12. Other studies have shown that IL-12 plays a role in protecting the host during *Leishmania* infection by promoting a Th1 response and controlling the parasitic replication [35–37]. Our data have also shown that melittin eliminated the intracellular amastigotes with an IC_50_ value about 20-fold smaller than that needed for the extracellular promastigotes, suggesting the participation of macrophages in its lethal effect.

TNF-α is a pro-inflammatory cytokine involved in the activation of macrophages and, together with IFN-γ, contributes to antiparasitic activity [38]. Although in our study melittin induced a significant decrease of TNF-α levels in infected macrophages, an effective antileishmanial activity was observed after melittin treatment in macrophages. Considering that an excess of TNF-α in the spleen has been described to contribute to a progressive cellular damage and also immunological dysfunction [39, 40], the observed downregulation of TNF-α induced by melittin could be a possible advantage to a future experimental study.

The cytokines IL-10 and TGF-β have been involved in homeostatic mechanisms of leishmaniasis by limiting the tissue damage caused by excessive inflammation. However, elevated levels of these cytokines have also been ascribed to the persistence of the infection [41]. It has also been reported that patients with active VL present high serum IL-10 levels prior to treatment, indicating their association with disease persistence [42, 43]. According to our results, the interaction of melittin with infected macrophages, decreased the IL-10 production when compared to untreated cells. Taken together, these findings suggest that the antiparasitic activity (of melittin) may also be ascribed to the upregulation of IL-12 levels and the downregulation of IL-10 levels, resulting in a reduced *in vitro* infection. Conversely, melittin did not exert an effect on TGF-β levels, and augmented the production of IL-10 in uninfected macrophages. These results are in agreement with the observations of Lapara and Kelly [44], who verified an increase in IL-10 production in macrophages infected by *Leishmania*.

Nitric oxide (NO) and hydrogen peroxide (H_2_O_2_) perform a fundamental role in the defense of macrophages [45–47]. In our assays, elevated levels of NO and H_2_O_2_ were found in *Leishmania*-infected macrophages, but upon treatment with melittin, a strong inhibition of these metabolites was observed. These observations corroborates Kwon *et al.* [48] and Moon *et al.* [49], who demonstrated the capacity of melittin to inhibit NO production. The elimination of intracellular amastigotes via an NO-independent pathway has also been described by Costa-Silva *et al.* [50]. The authors demonstrated the antileishmanial activity of a natural phenylpropanoid dimer on *L. donovani* macrophage infection, with similar diminutions in IL-10 levels.

## Conclusions

The results showed that melittin exerts *in vitro* activity against *Leishmania (L.) infantum* promastigotes and intracellular amastigotes and can act indirectly on intracellular amastigotes through a macrophage immunomodulatory effect.

### Ethics committee approval

The present study was approved by the Ethics Committee on Animal Experimentation of the Botucatu Medical School, São Paulo State University (UNESP), Botucatu, SP, Brazil, under protocol number CEEA 8932011 on 07/28/2011. Moreover, animal procedures were performed in agreement with the Guide for the Care and Use of Laboratory Animals from the National Academy of Sciences.

## References

[1] Alvar J, Vélez ID, Bern C, Herrero M, Desjeux P, Cano J (2012). Leishmaniasis worldwide and global estimates of its incidence. PLoS One.

[2] Allahverdiyev AM, Abamor ES, Bagirova M, Baydar SY, Ates SC, Kaya F (2013). Investigation of antileishmanial activities of Tio2@Ag nanoparticles on biological properties of *L. tropica* and *L. infantum* parasites, *in vitro*. Exp Parasitol.

[3] Cota GF, de Sousa MR, Demarqui FN, Rabello A (2012). The diagnostic accuracy of serologic and molecular methods for detecting visceral leishmaniasis in HIV infected patients: meta-analysis. PLoS Negl Trop Dis.

[4] Sundar S, Chakravarty J (2015). An update on pharmacotherapy for leishmaniasis. Expert Opin Pharmacother.

[5] Ferreira AS, Barraviera B, Barraviera SR, Abbade LP, Caramori CA, Ferreira Junior RS (2013). A success in toxinology translational research in Brazil: bridging the gap. Toxicon.

[6] Marques N, Cabral S, Sá R, Coelho F, Oliveira J, da Cunha JG S (2007). Leishmaniose visceral e infecção por vírus da imunodeficiência humana na era da terapêutica anti-retrovírica de alta eficácia. Acta Med Port.

[7] Adade CM, Chagas GS, Souto-Padrón T (2012). *Apis mellifera* venom induces different cell death pathways in *Trypanosoma cruzi*. Parasitology.

[8] Chen L, Chen W, Yang H, Lai R (2010). A novel bioactive peptide from wasp venom. J Venom Res.

[9] Carter V, Underhill A, Baber I, Sylla L, Baby M, Larget-Thiery I (2013). Killer bee molecules: antimicrobial peptides as effector molecules to target sporogonic stages of *Plasmodium*. PLoS Pathog.

[10] Jacobs T, Bruhn H, Gaworski I, Fleischer B, Leippe M (2003). NK-lysin and its shortened analog NK-2 exhibit potent activities against *Trypanosoma cruzi*. Antimicrob Agents Chemother.

[11] Conlon JM, Kolodziejek J, Nowotny N (2004). Antimicrobial peptides from ranid frogs: taxonomic and phylogenetic markers and a potential source of new therapeutic agents. Biochim Biophys Acta.

[12] Ferre R, Melo MN, Correia AD, Feliu L, Bardají E, Planas M (2009). Synergistic effects of the membrane actions of cecropin-melittin antimicrobial hybrid peptide BP100. Biophys J.

[13] Stromstedt AA, Wessman P, Ringstad L, Edwards K, Malmsten M (2007). Effect of lipid headgroup composition on the interaction between melittin and lipid bilayers. J Colloid Interface Sci.

[14] Kulkarni MM, McMaster WR, Kamysz W, McGwire BS (2009). Antimicrobial peptide-induced apoptotic death of *Leishmania* results from calcium-dependent, caspase-independent mitochondrial toxicity. J Biol Chem.

[15] Alberola J, Rodríguez A, Francino O, Roura X, Rivas L, Andreu D (2004). Safety and efficacy of antimicrobial peptides against naturally acquired Leishmaniasis. Antimicrob Agents Chemother.

[16] Ferrreira Junior RS, Sciani JM, Marques-Porto R, Lourenço Junior A, Orsi RO, Barraviera B (2010). Africanized honey bee (*Apis mellifera*) venom profiling: seasonal variation of melittin and phospholipase A2 levels. Toxicon.

[17] Santos LD, Pieroni M, Menegasso ARS, Pinto JRAS, Palma MS (2011). A new scenario of bioprospecting of Hymenoptera venoms through proteomic approach. J Venom Anim Toxins incl Trop Dis.

[18] Sciani JM, Marques-Porto R, Lourenço Junior A, Orsi RO, Ferreira Junior RS, Barraviera B (2010). Identification of a novel melittin isoform from Africanized *Apis mellifera* venom. Peptides.

[19] Cezário GAC, de Oliveira LR, Peresi E, Nicolete VC, Polettini J, de Lima CR (2011). Analysis of the expression of toll-like receptors 2 and 4 and cytokine production during experimental *Leishmania chagasi* infection. Mem Inst Oswaldo Cruz.

[20] Tempone AG, Pimenta DC, Lebrun I, Sartorelli P, Taniwaki NN, de Andrade HF J (2008). Antileishmanial and antitrypanosomal activity of bufadienolides isolated from the toad *Rhinella jimi* parotoid macrogland secretion. Toxicon.

[21] Reimão JQ, Colombo FA, Pereira-Chioccola VL, Tempone AG (2011). *In vitro* and experimental therapeutic studies of the calcium channel blocker bepridil: detection of viable *Leishmania* (*L*.) *chagasi* by real-time PCR. Exp Parasitol.

[22] Tada H, Shiho O, Kuroshima K, Koyama M, Tsukamoto M (1986). An improved colorimetric assay for interleukin 2. J Immunol Methods.

[23] Griess P (1879). Bemerkungen zu der abhandlung der HH. Weselsky und Benedikt, Ueber einige Azoverbindungen. Ber Dtsch Chem Ges.

[24] Pick E, Keisari Y (1980). A simple colorimetric method for the measurement of hydrogen peroxide produced by cells in culture. J Immunol Methods.

[25] Pick E, Mizel D (1981). Rapid microassays for the measurement of superoxide and hydrogen peroxide production by macrophages in culture using an automatic enzyme immunoassay reader. J Immunol Methods.

[26] Zar JH (2010). Biostatistical analysis.

[27] Bechinger B, Lohner K (2006). Detergent-like actions of linear amphipathic cationic antimicrobial peptides. Biochim Biophys Acta.

[28] Klocek G, Seelig J (2008). Melittin interaction with sulfated cell surface sugars. Biochemistry.

[29] Maher S, McClean S (2006). Investigation of the cytotoxicity of eukaryotic and prokaryotic antimicrobial peptides in intestinal epithelial cells *in vitro*. Biochem Pharmacol.

[30] Díaz-Achirica P, Ubach J, Guinea A, Andreu D, Rivas L (1998). The plasma membrane of *Leishmania donovani* promastigotes is the main target for CA(1–8)M(1–18), a synthetic cecropin A-melittin hybrid peptide. Biochem J.

[31] Petricevich VL, Teixeira CF, Tambourgi DV, Gutiérrez JM (2000). Increments in serum cytokine and nitric oxide levels in mice injected with *Bothrops asper* and *Bothrops jararaca* snake venoms. Toxicon.

[32] Pérez-Santos JL, Talamás-Rohana P (2001). *In vitro* indomethacin administration upregulates interleukin-12 production and polarizes the immune response towards a Th1 type in susceptible BALB/c mice infected with *Leishmania mexicana*. Parasite Immunol.

[33] Passero LF, Laurenti MD, Tomokane TY, Corbett CE, Toyama MH (2008). The effect of phospholipase A2 from *Crotalus durissus collilineatus* on *Leishmania* (*Leishmania*) *amazonensis* infection. Parasitol Res.

[34] Mukbel RM, Patten C, Gibson K, Ghosh M, Petersen C, Jones DE (2007). Macrophage killing of *Leishmania amazonensis* amastigotes requires both nitric oxide and superoxide. Am J Trop Med Hyg.

[35] Hernandez-Pando R, Orozco H, Arriaga K, Sampieri A, Larriva-Sahd J, Madrid-Marina V (1997). Analysis of the local kinetics and localization of interleukin-1 alpha, tumour necrosis factor-alpha and transforming growth factor-beta, during the course of experimental pulmonary tuberculosis. Immunology.

[36] Watford WT, Hissong BD, Bream JH, Kanno Y, Muul L, O’Shea JJ (2004). Signaling by IL-12 and IL-23 and the immunoregulatory roles of STAT4. Immunol Rev.

[37] Cummings HE, Tuladhar R, Satoskar AR (2010). Cytokines and their STATs in cutaneous and visceral leishmaniasis. J Biomed Biotechnol.

[38] Coelho-Castelo AAM, Trombone APF, Rocha CD, Lorenzi JCC (2009). Resposta imune a doenças infecciosas. Medicina (Ribeirão Preto).

[39] Robak T, Gladalska A, Stepien H (1998). The tumour necrosis factor family of receptors/ligands in the serum of patients with rheumatoid arthritis. Eur Cytokine Netw.

[40] Bradley JR (2008). TNF-mediated inflammatory disease. J Pathol.

[41] Belkaid Y, Hoffmann KF, Mendez S, Kamhawi S, Udey MC, Wynn TA (2001). The role of interleukin (IL)-10 in the persistence of *Leishmania major* in the skin after healing and the therapeutic potential of anti-IL-10 receptor antibody for sterile cure. J Exp Med.

[42] Ansari NA, Saluja S, Salotra P (2006). Elevated levels of interferon-gamma, interleukin-10, and interleukin-8 during active disease in Indian kala azar. Clin Immunol.

[43] Verma S, Kumar R, Katara GK, Singh LC, Negi NS, Ramesh V (2010). Quantification of parasite load in clinical samples of leishmaniasis patients: IL-10 level correlates with parasite load in visceral leishmaniasis. PLoS One.

[44] Lapara NJ, Kelly BL (2010). Suppression of LPS-induced inflammatory responses in macrophages infected with *Leishmania*. J Inflamm (Lond).

[45] Bogdan C, Mayer B (2000). The function of nitric oxide in the immune system. Nitric Oxide.

[46] Tripathi P, Tripathi P, Kashyap L, Singh V (2007). The role of nitric oxide in inflammatory reactions. FEMS Immunol Med Microbiol.

[47] Barros GAC, Pereira AV, Barros LC, Lourenço A, Calvi SA, Santos LD (2015). *In vitro* activity of phospholipase A_2_ and of peptides from *Crotalus durissus terrificus* venom against amastigote and promastigote forms of *Leishmania (L.) infantum chagasi*. J Venom Anim Toxins incl Trop Dis.

[48] Kwon YB, Kang MS, Kim HW, Ham TW, Yim YK, Jeong SH (2001). Antinociceptive effects of bee venom acupuncture (apipuncture) in rodent animal models: a comparative study of acupoint versus non-acupoint stimulation. Acupunct Electrother Res.

[49] Moon DO, Park SY, Lee KJ, Heo MS, Kim KC, Kim MO (2007). Bee venom and melittin reduce proinflammatory mediators in lipopolysaccharide-stimulated BV2 microglia. Int Immunopharmacol.

[50] da Costa-Silva TA, Grecco SS, de Sousa FS, Lago JH, Martins EG, Terrazas CA (2015). Immunomodulatory and antileishmanial activity of phenylpropanoid dimers isolated from *Nectandra leucantha*. J Nat Prod.

